# Untangling the Gordian knot—further resolving the super-species complex of 300-million-year-old xiphosurids by reconstructing their ontogeny

**DOI:** 10.1007/s00427-020-00648-7

**Published:** 2020-01-31

**Authors:** Carolin Haug, Joachim T. Haug

**Affiliations:** 1 Department of Biology II, LMU Munich, Biocenter, Großhaderner Str. 2, 82152 Planegg-Martinsried, Germany; 2 GeoBio-Center of the LMU Munich, Richard-Wagner-Str. 10, 80333 Munich, Germany

**Keywords:** *Euproops*, *Belinurus*, Heterochrony, Metamorphosis, Fossilised ontogeny

## Abstract

The group Xiphosurida (horseshoe “crabs”) is today only represented by four species. However, in the fossil record, several dozen species have been described, especially from the Carboniferous (about 300 million years ago). Several species have been interpreted as representatives of *Euproops* or *Belinurus*, but there is ongoing discussion which of these species are valid and how they can be differentiated. Recent studies suggested that differences in the timing of individual development could provide information for species distinction, exemplified by studies on *Euproops danae* (Mazon Creek, USA) and *Euproops* sp. (“Piesproops”; Piesberg, Germany). For this study, we reinvestigated all Carboniferous xiphosurids from the British Coal Measures stored in the collections of the Natural History Museum London. Size comparisons of the specimens revealed nine size groups; the smaller specimens were originally labelled as *Belinurus*, the larger ones as *Euproops*. The nine size groups exhibit five different morphotypes differing in structures surrounding the posterior shield (= thoracetron): spines of different lengths and, in larger specimens, a more or less developed flange. Two of these morphotypes show significantly longer spines than the remaining specimens and could be conspecific as *E. anthrax*. The remaining specimens are interpreted as growth series of another species, presumably of *E. rotundatus*. An ontogenetic flange formation is also known from *E. danae* and the “Piesproops”, but the timing differs between all three species. In *E. rotundatus*, the flange develops rather late, but then comparably abruptly, which makes this development more metamorphic in relation to development in the other species.

## Introduction

Xiphosurids, commonly referred to as horseshoe “crabs”, are not (as their name implies) crustaceans, but representatives of Euchelicerata, hence closer relatives of scorpions, spiders and the like. The now only rarely used term “sword tail” is therefore more appropriate, yet unfortunately also used for fishes of the species *Xiphophorus*. Xiphosurids are often treated as being something ancient (e.g. Sekiguchi 1988; Malakhov 2010; Williams 2019) and used as a kind of direct proxy for the stem species (“ancestor”) of Euchelicerata (e.g. Battelle et al. 2016). Yet, modern xiphosurids evolved their own specialisations and are not direct ancestors of the remaining forms of Euchelicerata.

Xiphosurids are nowadays only represented by four extant species, but the lineage appears to have been more species-rich in the past (e.g. Anderson and Selden 1997; Lamsdell 2016). Especially from the Carboniferous, roughly 300 million years ago, numerous species have been formally named and described. More than half a dozen species groups (traditional genera) are known from this time. Most widely known are species of *Belinurus* and *Euproops*.

As an aside, the name *Belinurus* is used here in the spelling with one l, in contrast to the spelling *Bellinurus*, which is based on Pictet (1846). Several authors cite Pictet (1846) as first mentioning of the name, although Pictet cites König as the source, yet without the year. Dunlop et al. (2019) state: “Pictet’s 1846 name *Bellinurus* [sic] was based on a misspelling of *Belinurus* from König’s unpublished plates, which themselves only became available posthumously as of 1851”. However, the work of König has apparently already been published in 1820–1825, according to the information provided by the Biodiversity Heritage Library, probably based on information from the NHM London as holding institution of König’s work (there is no publication year written on the work itself). In König’s work, the spelling *Belinurus* is used for fig. 230 on Plate XVIII (yet, the main text only describes the figures up to fig. 100). Therefore, *Belinurus* has priority over the misspelled *Bellinurus* and is used in the following.

While there were more fossil xiphosurid species than extant ones, the number of Carboniferous species has dropped after Anderson (1994) synonymised most species of the group *Euproops* into the single species *Euproops danae*. Other authors have addressed the problem of identifying valid species within *Euproops*, i.e. whether there is one versus several species, as the “*Euproops danae*-*rotundatus* species complex” (Schultka 2000). This expression indicates that the two species *E. danae* and *E. rotundatus* are not extremely similar, but also not perfectly distinct, with certain intermediate forms linking these two supposedly different ones.

The two species *E. rotundatus* and *E. danae* indeed appear to differ in the morphology of the armature of their thoracetron (“opisthosomal shield”, the dorsal sclerotisation formed by the posterior body region): The one species, *E. danae* from the Mazon Creek formation, USA, bears numerous curved spines; the other species, *E. rotundatus*, from the British Coal Measures bears plates forming a surrounding flange (see discussion in Haug et al. 2012).

This distinction might appear simple, yet the situation between these two species is in fact significantly more complicated. Contemporary specimens of *Euproops* sp. from Germany allowed the reconstruction of an ontogenetic sequence of ten subsequent stages (Haug et al. 2012), showing that this species changes from a morphology resembling *E. danae* (with spines) over intermediate forms to forms resembling *E. rotundatus* (with a flange formed by plates).

This finding not only complicates the distinction between the two species of *Euproops*. It also demonstrates that, although representatives of Euchelicerata are generally considered to hatch with a very similar morphology as the corresponding adult, their morphology can change quite significantly during post-embryonic ontogeny. The reconstruction of the ontogenetic sequence of the species from Germany prompted a reinvestigation of *E. danae* and *E. rotundatus* with a focus on details of their ontogeny. The ontogenetic sequence of *E. danae* was recently reconstructed by Haug and Rötzer (2018). Of additional material from Windber, PA, USA, assumed to be also *E. danae* further aspects of morphological variation in the ontogeny were described by Tashman et al. (2019). Here we provide a reinvestigation of xiphosurids from the British Coal Measures, including the specimens traditionally considered to represent *E. rotundatus*, with the focus on identifying possible ontogenetic changes.

## Material and methods

### Material

All specimens in this study are housed in the Natural History Museum, London. We inspected all specimens that were labelled as Xiphosura found in the British Coal Measures and are hence originating from Carboniferous strata.

### Documentation methods

All specimens were documented for two different aspects, colour information and three-dimensional relief information. Due to the special preservation of Coal Measures fossils preserved in nodules, this avoids artefacts that might be caused by misinterpreting colour information as relief information (i.e. darker coloured areas could be misinterpreted as shadows). Examples for the principle approach have been presented in Haug et al. (2012, 2015, 2016).

Photos were recorded with a Canon EOS Rebel T3i equipped with an MP-E 65-mm macro lens. Illumination was provided by a Canon MT-24EX twin flash.

For documenting colour information, specimens were evenly illuminated avoiding shadows. Cross-polarised light was used to further extinguish directed light from the surroundings and to enhance the colour contrast (e.g. Haug et al. 2011; Kerp and Bomfleur 2011). Images were recorded as a stack of frames of shifting focus in cases in which the depth of field was insufficient to result in a fully sharp image. Stacks were fused using CombineZP (for details, see Haug et al. 2008, 2011). Further optimising was performed in Adobe Photoshop CS2 (levels, saturation, sharpness). Levels were optimised for each colour channel separately. Unnaturally appearing blueish areas occur as a side effect of the cross-polarised light. Saturation of blue and cyan was therefore decreased.

For documenting relief, a series of images of the same specimen was recorded under different angles of view. Images were desaturated and assembled to red-cyan stereo images (e.g. Haug et al. 2012). Image pairs were chosen to provide sufficient depth impression that can still be viewed without inducing interference. Rather flat relief may therefore appear stronger elevated in the images. Some stereo images of counterparts were depth inverted to reveal the original relief.

## Results

### Included specimens

The material investigated, xiphosurids from the British Coal Measures, included three distinct traditionally recognised morphotypes that could in fact be further differentiated into more types (see below). The three traditional types correspond to the three traditional species groups (“genera”) *Liomesaspis*, *Euproops*, and *Belinurus*. Only specimens that fall into the *Belinurus* group and *Euproops* group were further considered here. Specimens of the *Liomesaspis* group differ strongly from the other specimens by being highly vaulted and lacking prominent structures around the thoracetron.

### Recognising size groups

**Fig. 1:**
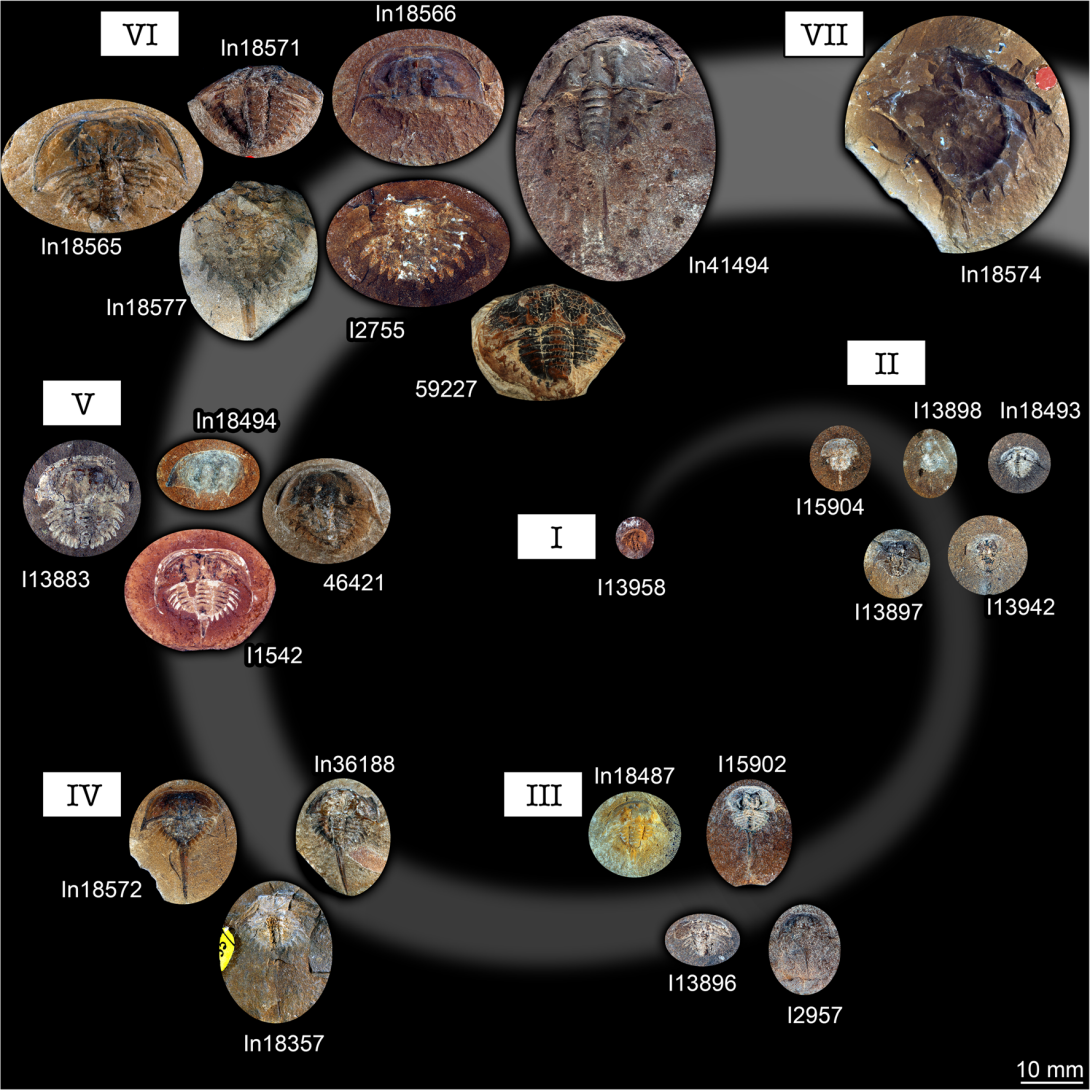
Xiphosurids from the Carboniferous British Coal Measures. Specimens of unclear type, type 1, and type 2 arranged as growth series, continued in Fig. 2. All specimens falling into size groups 1–6 have originally been labelled as representatives of *Belinurus*, while the specimen falling into size group 7 has originally been labelled as *Euproops danae* (and *Prestwichia rotundata* and *Prestwichianella rotundata*, which are all synonyms)

**Fig. 2:**
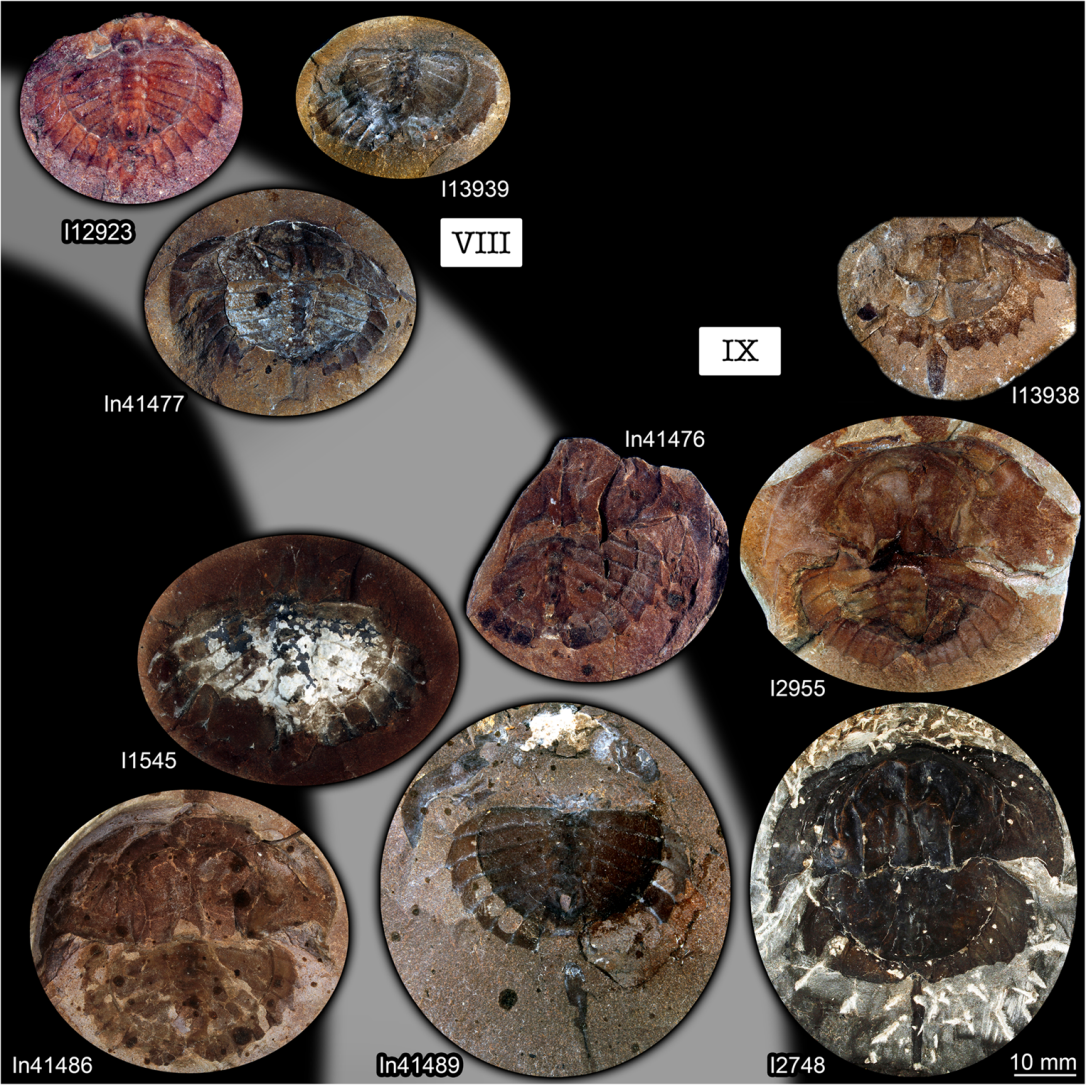
Xiphosurids from the Carboniferous British Coal Measures, continued. Specimens of type 3 arranged as growth series, continued from Fig. 1. All specimens falling into size groups 8 and 9 have originally been labelled as representatives of *Euproops* (or *Prestwichia* and *Prestwichianella*, which are all synonyms)

In studies of fossils of the group *Euproops* from Germany (“Piesproops”; Haug et al. 2012) and the USA (*E. danae*; Haug and Rötzer 2018), the material was grouped into size groups using measurements of discrete dimensions. This strategy proved difficult to apply to the material from the British Coal Measures. Too many specimens were incomplete or distorted in the one or other dimension to acquire a stable dataset. Instead, images of all specimens were re-sized to the same scale. Then specimens were compared by overlaying them digitally. Specimens with roughly the same size were grouped together. Nine size groups could be recognised in this way (Figs. 1 and 2).

### Overall morphology

The overall morphology is very similar in all specimens (Figs. 1 and 2). More complete specimens possess two distinct dorsal sclerotisations of the body. The anterior body forms an anterior shield (“prosomal shield”), the posterior region, the trunk, forms the thoracetron. In some specimens, an articulated spine-like “tail” emerges from the trunk, the telson. Some specimens consist only of an isolated anterior shield or thoracetron.

The anterior shield appears highly variable in shape, strongly depending on the angle of embedding. In well-preserved specimens, the principal shape is well apparent. The anterior edge is gently rounded, curving backwards until meeting with the posterior edge. The posterior edge appears to have been more or less straight in exact dorsal view. Yet, the anterior shield was apparently arched in dorso-ventral axis. Depending on the exact angle of embedding, the posterior edge therefore can appear concave (if seen a bit more from posterior) or slightly convex (if seen a bit more from anterior). The postero-lateral edges are drawn out into prominent spines (genal spines). A prominent ridge (opthalmic ridge) is apparent in many specimens, to varying degree. The original presence can be assumed for all specimens. The ridge is heart-shaped anteriorly, but does not converge into a single point but runs straight backwards until meeting with the posterior edge. In some specimens, possible compound eyes are apparent on this ridge. In an isolated anterior shield (In18494, Fig. 1), the ophthalmic ridges continue into spines. These appear to be originally present, but broken off in most specimens.

The thoracetron morphology is more variable. Similar in all specimens is the fact that it is approximately semi-circular with a straight anterior edge, but with a certain shape variation (see also next paragraph). Dorsally, the thoracetron bears prominent grooves as indications of segmentation. At least eight such segments are apparent. These segments become more U-shaped towards the posterior. The median region is differentiated as a distinct elevated axis. The grooves indicating the segments are also continuing beyond the axis. Additional structures (epimera) surround the thoracetron. These structures exhibit the most prominent variation, explained in detail below.

### Recognised thoracetron morphologies

The overall shape of the thoracetron seems not to be an appropriate character for distinguishing types. There are more rounded and more triangular forms, in fact representing a more or less continuous gradient. Therefore, the main character distinguishing specific types is the armature of the thoracetron, the epimera. Five distinct types can be recognised in addition to an unclear type.

#### Unclear type

**Fig. 3:**
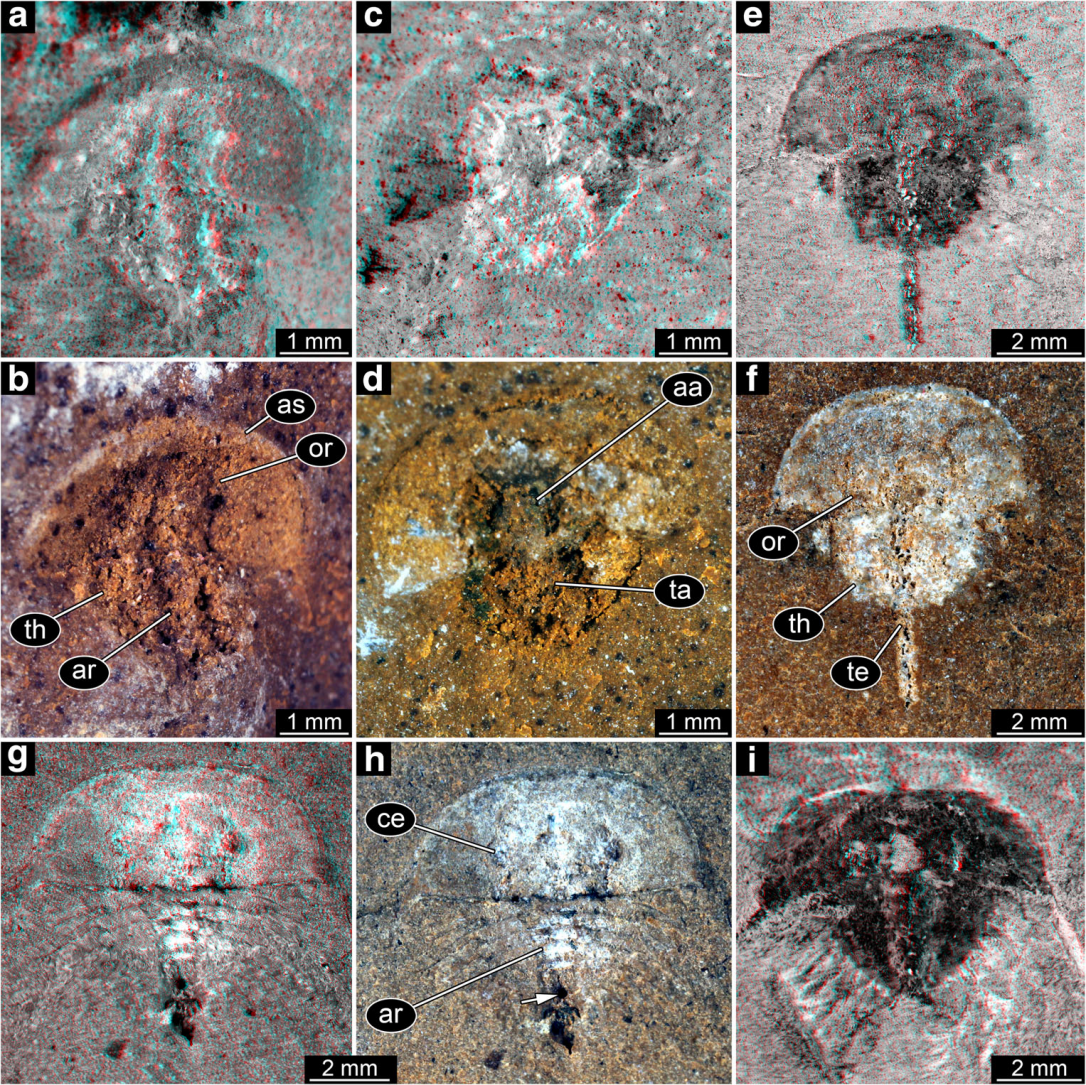
Size groups I and II (part). **a**–**d** Size group I; specimen I13958. **a**, **b** Part. **c**, **d** Counterpart. **e**–**i** Size group II. **e**, **f** Specimen I15904. **g**, **h** Specimen I13942. **h** Arrow points to spine (compare corresponding region in (**g**)). **i** Specimen I18493; note the anterior shield diving into the matrix. **a**, **c**, **e**, **g**, **i** Stereo anaglyphs (use red-cyan glasses to view). **e**, **g**, **i** Depth inverted. **b**, **d**, **f**, **h** Compound images under cross-polarised light. aa, anterior appendages; ar, axial region; as, anterior shield; ce, compound eye; or, ophthalmic ridge; ta, trunk appendages; te, telson; th, thoracetron

Size group 1, the smallest size group, is represented by a single specimen (Fig. 3a–d). It is not well preserved and can therefore not be identified to a specific type. As it is the smallest specimen, it is still considered important and included here.

#### Type 1

**Fig. 4:**
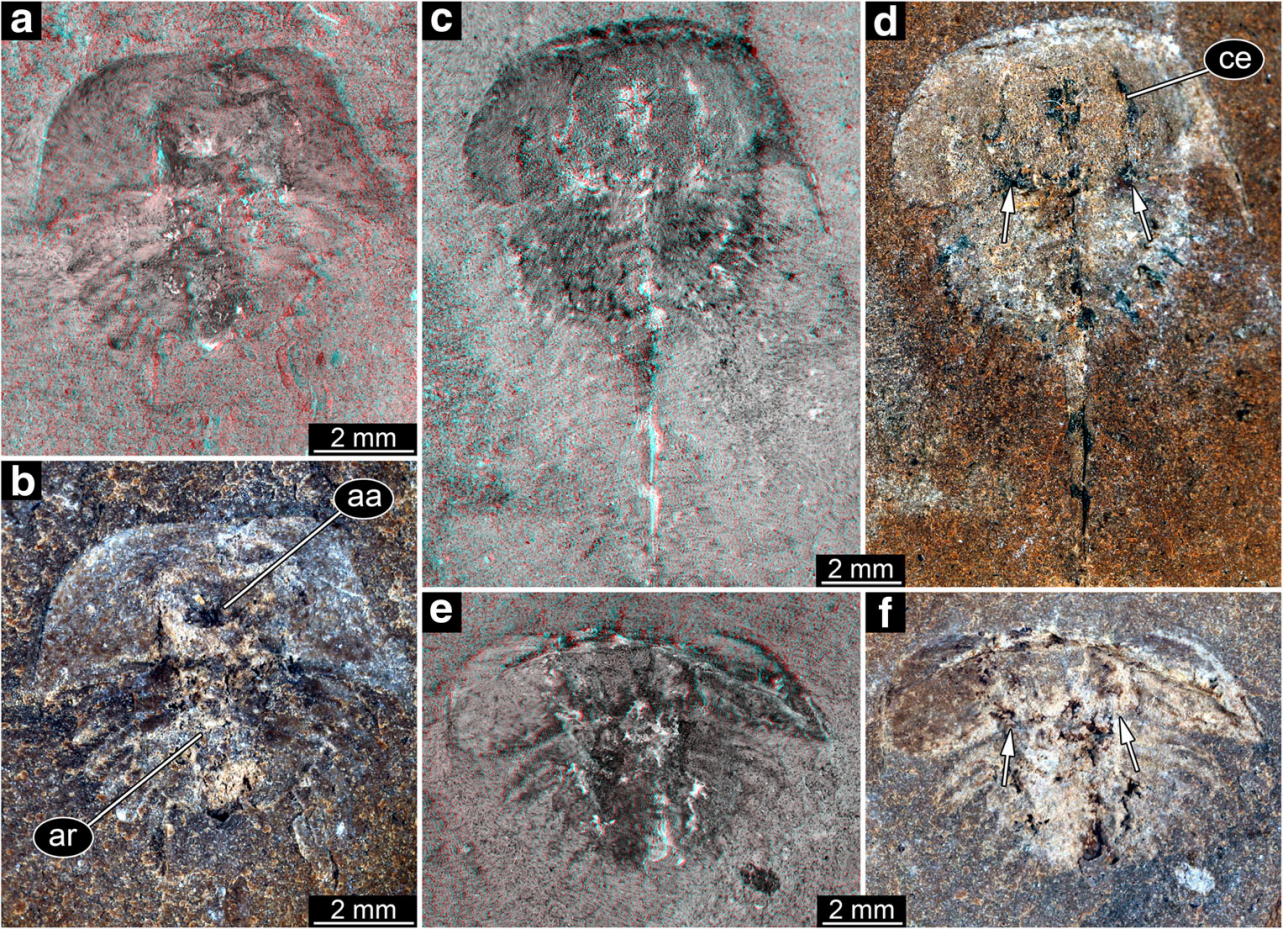
Size groups II (continued) and III. **a**, **b** Size group II; specimen I13897. **c**–**f** Size group III. **c**, **d** I15902. **e**, **f** I13896. **d**, **f** Arrows point to the bases of broken off ophthalmic spines. **a**, **c**, **e** Stereo anaglyphs (use red-cyan glasses to view). **e** Depth inverted. **b**, **d**, **f** Compound images under cross-polarised light. aa, anterior appendages; ar, axial region; ce, compound eye

**Fig. 5:**
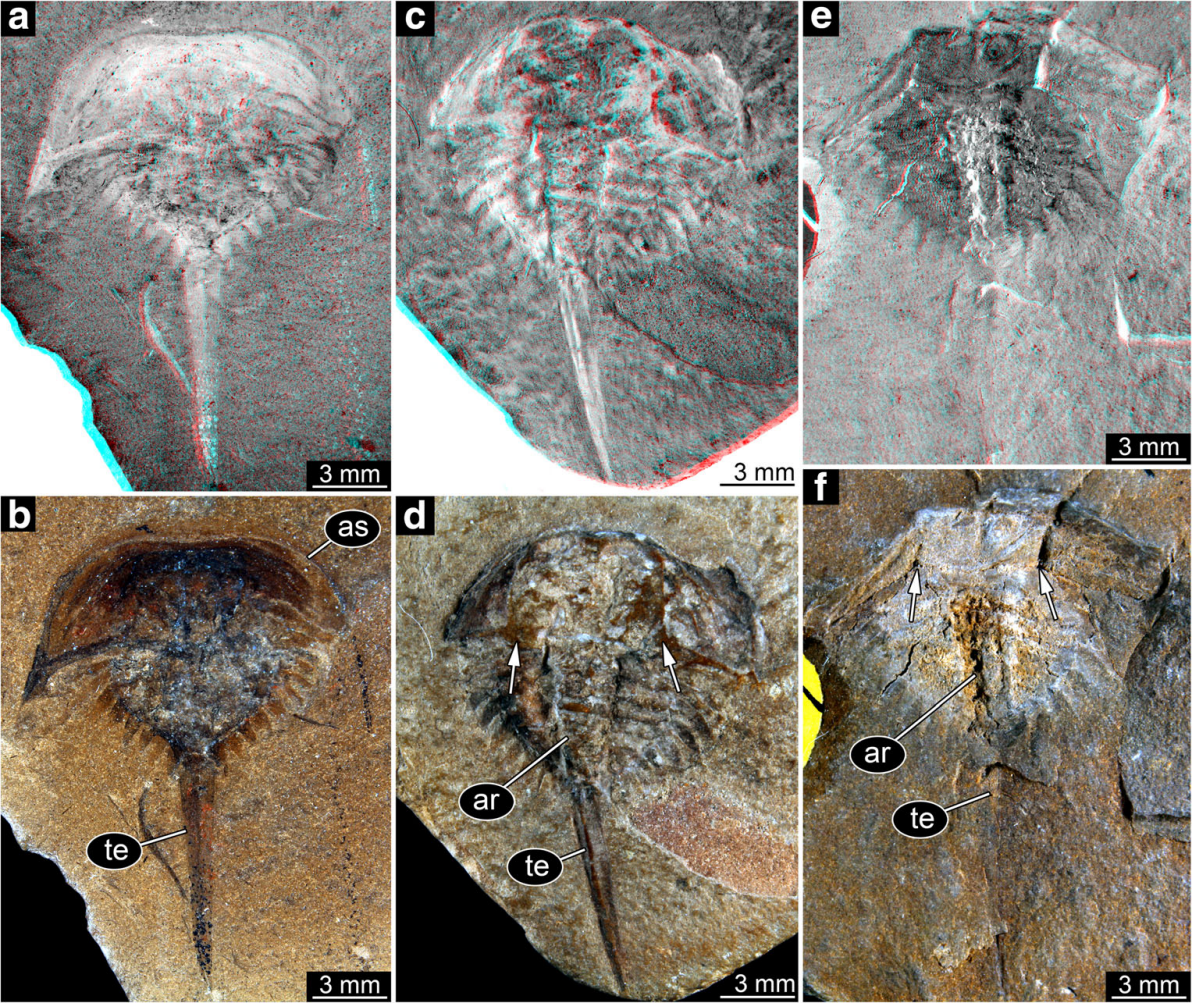
Size group IV. **a**, **b** In18572. **c**, **d** In36188. **e**, **f** In18357. **a**, **c**, **e** Stereo anaglyphs (use red-cyan glasses to view). **e** Depth inverted. **b**, **d**, **f** Compound images under cross-polarised light. **d**, **f** Arrows point to the bases of broken off ophthalmic spines. ar, axial region; te, telson

**Fig. 6:**
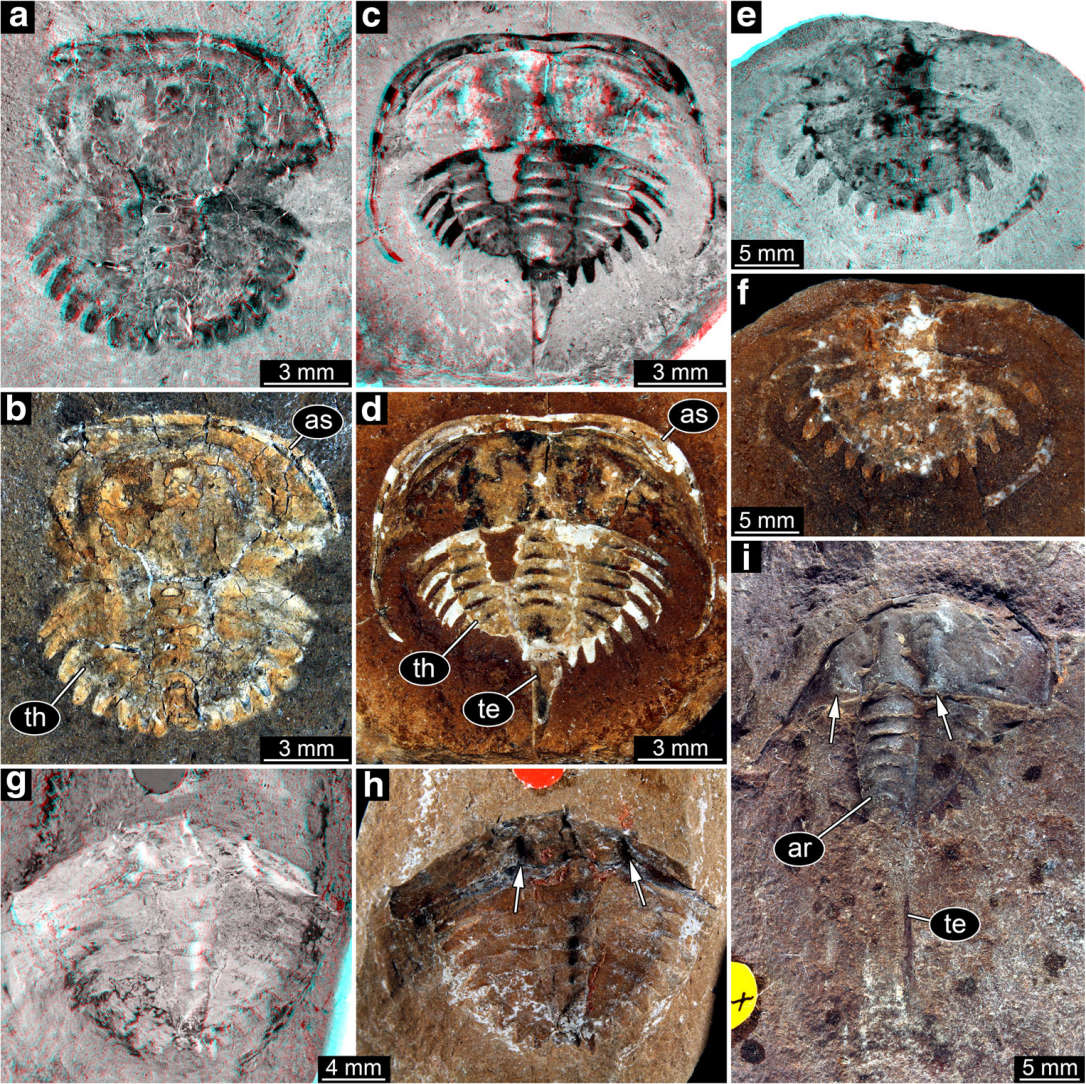
Size groups V and VI. **a**–**d** Size group V. **a**, **b** I13883. **c**, **d** I1542. **e**–**i** Size group VI. **e**, **f** I2755. **g**, **h** In18571; note the anterior shield diving into the matrix. **i** In41494. **a**, **c**, **e**, **g** Stereo anaglyphs (use red-cyan glasses to view). **a**, **g** Depth inverted. **b**, **d**, **f**, **h**, **i** Compound images under cross-polarised light. **h**, **i** Arrows point to the bases of broken off ophthalmic spines. ar, axial region; as, anterior shield; te, telson; th, thoracetron

The structures around the thoracetron are spines, eight on each side. The spines are very distinct. They all appear separated, not being partly continuous at their proximal region. Specimens of this type can be further differentiated into five size groups (size groups 2–6; Figs. 3e–i, 4, 5, and 6).

#### Type 2

**Fig. 7:**
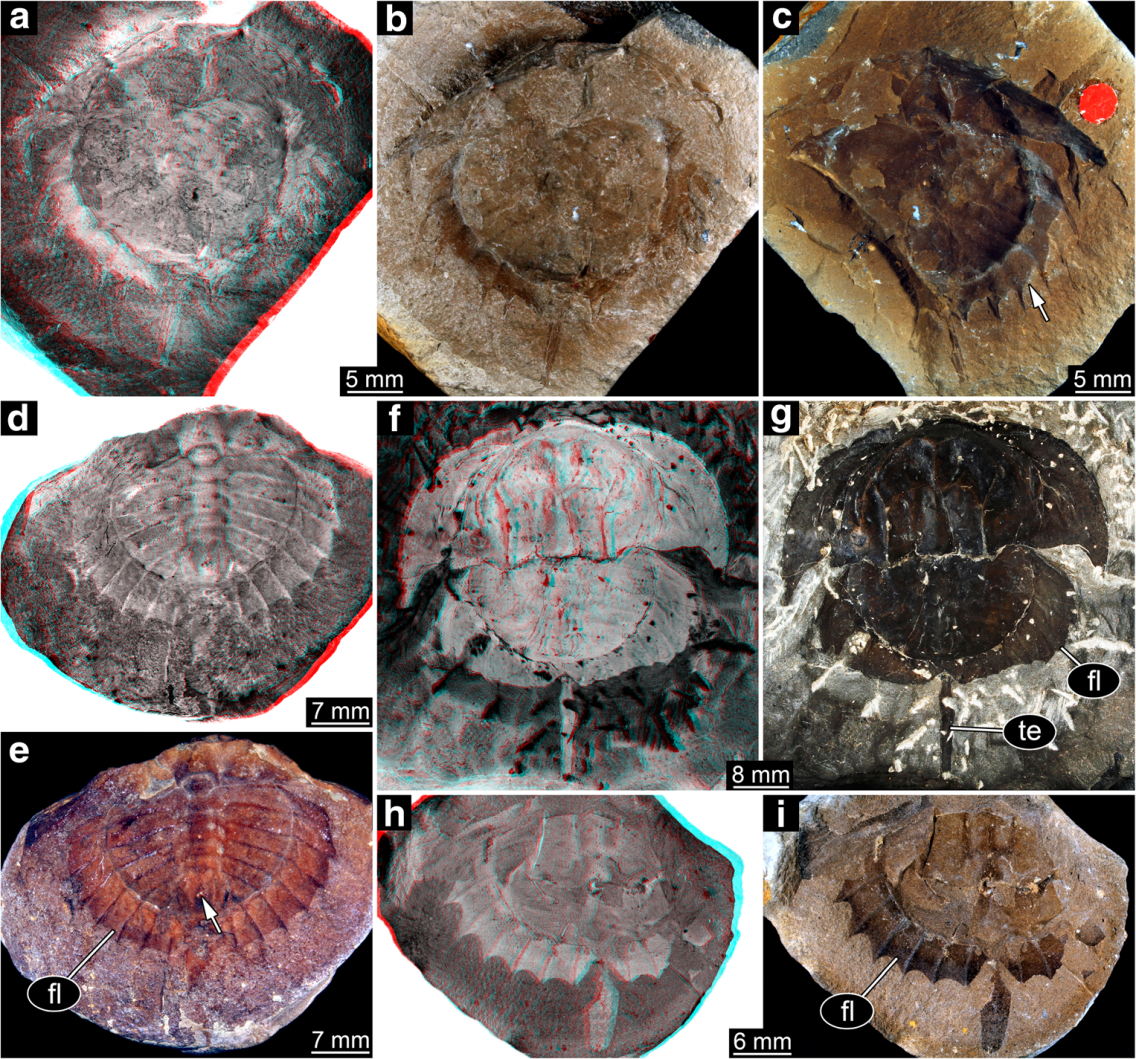
Size groups VII, VIII, and IX. **a**–**c** Size group VII; specimen In18574. **a**, **b** Part. **c** Counterpart; arrow points to half developed flange. **d**, **e** Size group VIII; I12923. **e** Arrow points to spine. **f**, **i** Size group IX. **f**, **g** I2748. **h**, **i** I13938. **a**, **d**, **f**, **h** Stereo anaglyphs (use red-cyan glasses to view). **b**, **c**, **e**, **g**, **i** Compound images under cross-polarised light. fl, flange; te, telson

Spines around the thoracetron are also distinct, but only distally. The proximal region of the spines forms a continuous flange around the thoracetron. This flange reaches about half the height of the spine, and the distal part of the spine is free. A single specimen represents this morphology, it is larger than the specimens of type 1 (size group 7; Fig. 7a–c).

#### Type 3

There are no true spines around the thoracetron. A continuous flange is present. The flange includes “ribs” that correspond to the position of spines in the other types. Specimens of this type are larger than the specimens of types 1 and 2 and fall into two separate size groups (size groups 8–9; Fig. 7d–i).

**Fig. 8:**
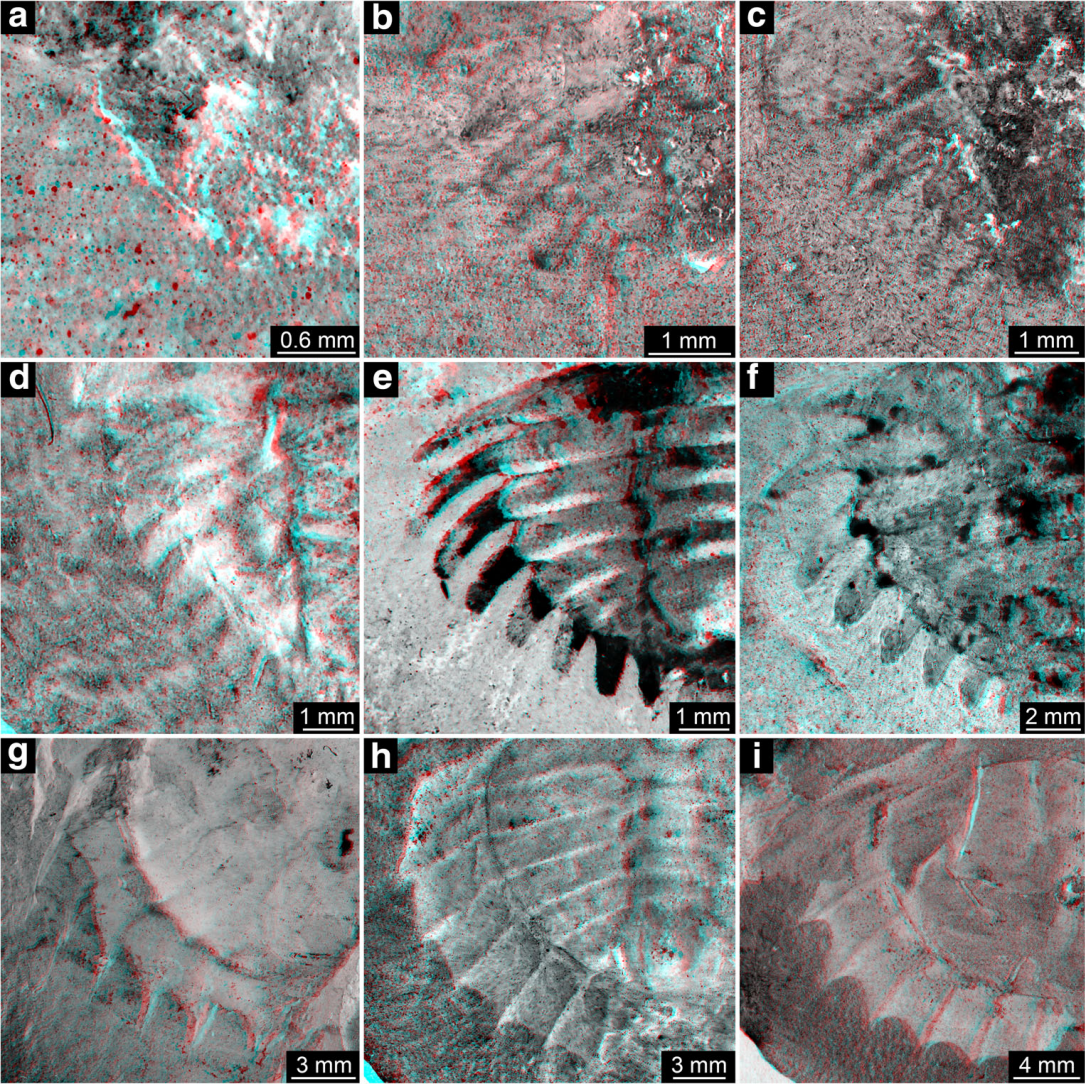
Flange morphologies of the size groups. **a** Size group I; I13958; flipped. **b** Size group II; I13897. **c** Size group III; I13896; depth inverted. **d** Size group IV; In36188. **e** Size group V; I1542; flipped. **f** Size group VI; I2755. **g** Size group VII; In18574; flipped. **h** Size group VIII; I12923; flipped. **i** Size group IX; I13938. **a**–**f** Without any flange. **g** With half developed flange. **h**, **i** With fully developed flange. All stereo anaglyphs (use red-cyan glasses to view)

The flange morphologies of the nine size groups are illustrated in Fig. 8.

#### Type 4

**Fig. 9:**
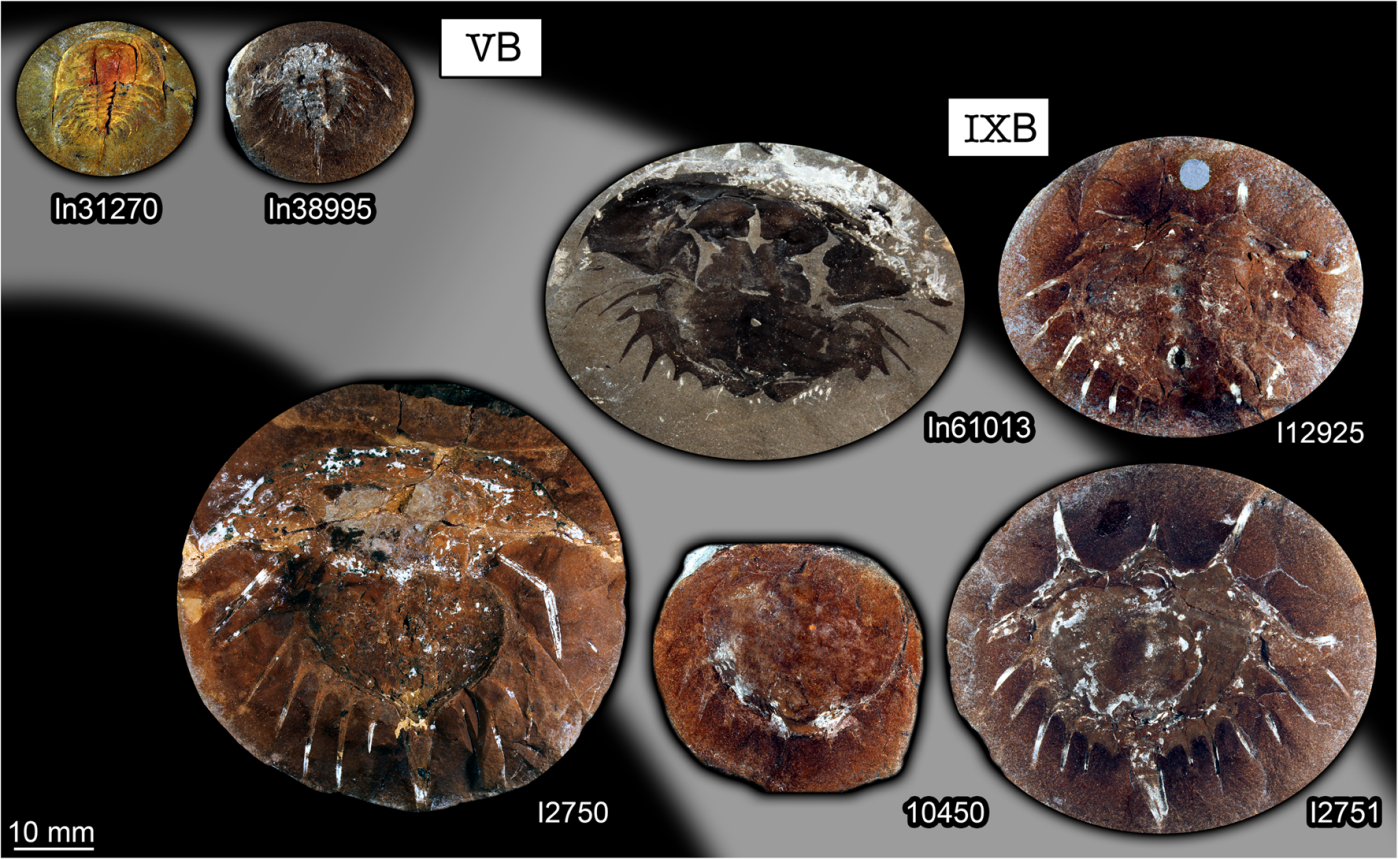
Xiphosurids from the Carboniferous British Coal Measures. Specimens of type 4 and 5 arranged as growth series. The specimens are originally labelled as follows: In31270, In38995: *Belinurus* (without species name); In61013: *Euproops danae* (previously *Euproops kilmersdonensis*); I12925, 10450, I2750, I2751: *Euproops anthrax* (I2750 previously *Prestwichia anthrax*)

**Fig. 10:**
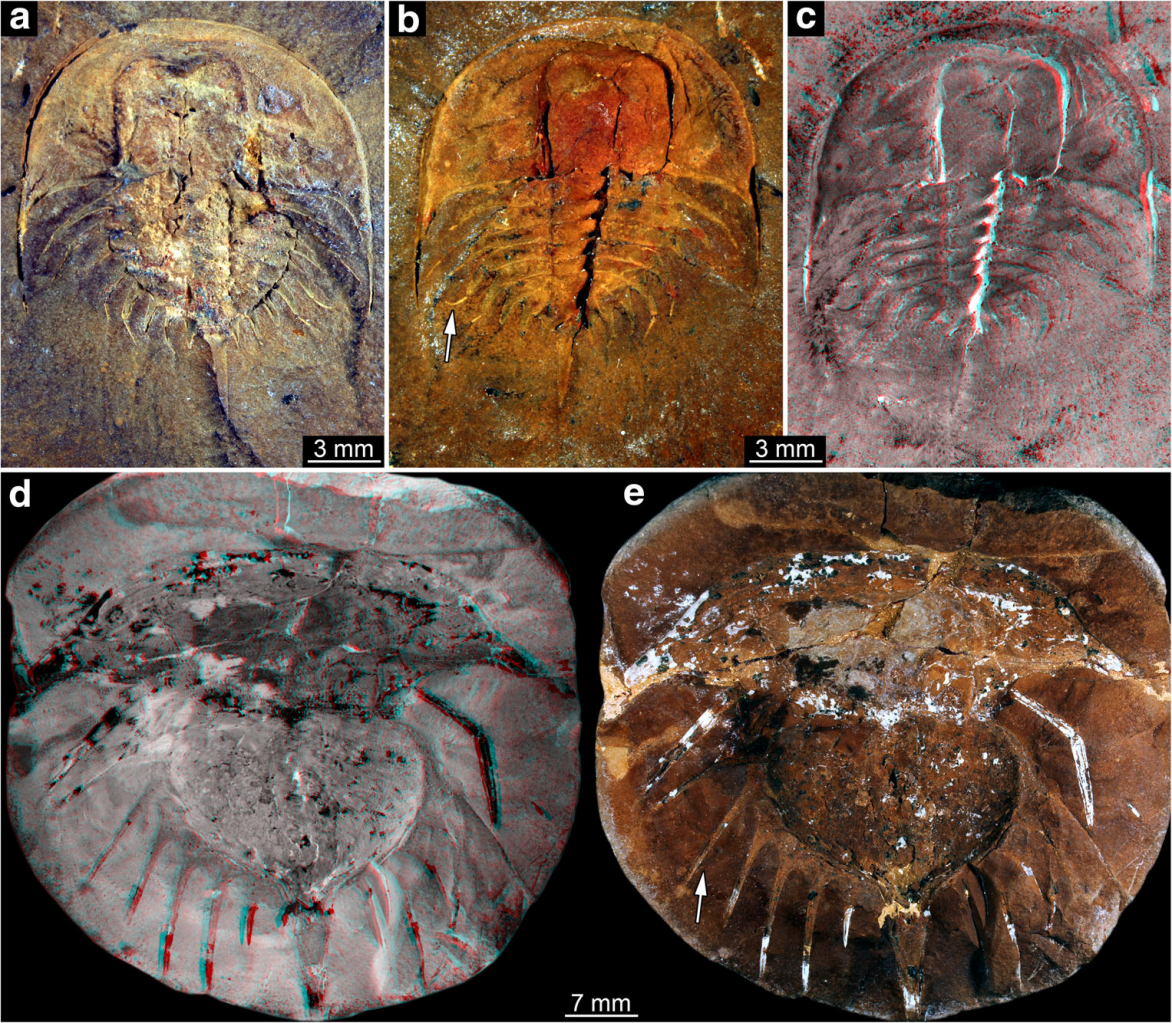
Size groups VB and IXB (part). **a**–**c** Size group VB; In31270. **a** Part. **b**, **c** Counterpart. **d**, **e** Size group IXB; I2750. **a**, **b**, **d** Compound images under cross-polarised light. **c**, **d** Stereo anaglyphs (use red-cyan glasses to view). **b**, **e** Arrows point to very long spines

**Fig. 11:**
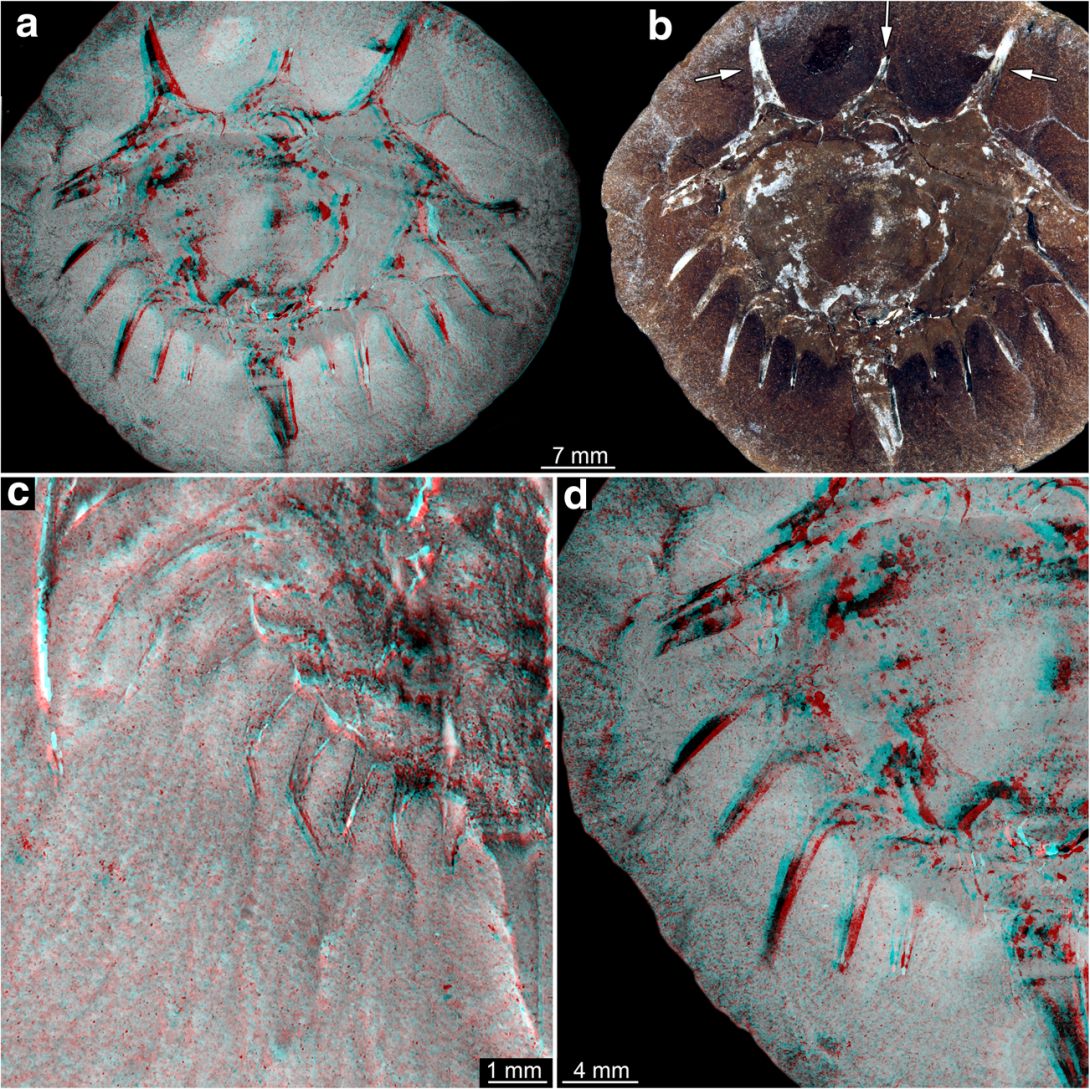
Size groups VB and IXB, continued. **a**, **b** Size group IXB; I2751; arrows point to ophthalmic and median spines. **c**, **d** Flange morphologies. **c** In31270; without any flange. **d** I2751; with partly developed flange. **a**, **c**, **d** Stereo anaglyphs (use red-cyan glasses to view). **b** Compound image under cross-polarised light

Spines around the thoracetron are distinct, comparable with type 1. Yet, the spines are significantly longer than in type 1 specimens. Only two specimens show this morphology, both correspond to the size group 5; to distinguish them, they are treated as group 5B (Figs. 9, 10a–c, and 11C).

#### Type 5

Spines around the thoracetron are present, but only distally. In this aspect, the specimens resemble the specimen representing type 2, and also here, a proximal flange is present. The flange is also comparable in size, yet in type 5 specimens, the distal part, i.e. the spines, is significantly longer. Also the specimens are significantly larger, corresponding to the size group 9 of type 3, hence referred to as size group 9B (Figs. 9, 10d, e, and 11a, b, d).

## Discussion

### Recognised size groups

It is unfortunate that the material from the British Coal Measures proved to be more difficult to measure than the specimens from Germany and the USA. This is most likely coupled to the sample size which was significantly smaller. Therefore, also specimens in a preservational state that would have been excluded in the other two studied forms had to be included here. Although the recognition of size groups is less distinct, we still see this as an acceptable proxy.

### Distinguishing *Euproops* from *Belinurus*

*Belinurus* is generally thought to lack pronounced ophthalmic spines (see e.g. Selden and Siveter 1987). Yet, already Fisher (1977) and Anderson (1994) have pointed out that in specimens of species of *Euproops*, these spines often remain hidden in the matrix of the counterpart (see also Haug and Rötzer 2018, their fig. 2B, C for an example). Indeed, specimens attributed to *Belinurus* also appear to possess these structures (Anderson and Selden 1997).

Also in the material investigated here, this becomes apparent. The ophthalmic ridges appear very strong towards the posterior rim and appear to form a basis for such spines (Figs. 4c–f, 5c–f, and 6g–i), hence indicating the original presence of these spines. Additionally, spines can be found on isolated anterior shields that cluster with specimens originally interpreted as *Belinurus*-type.

Another factor of discrimination of *Belinurus* from *Euproops* appears to have been the shape of the ophthalmic ridges in dorsal view. These are generally reconstructed straight for specimens attributed to *Belinurus* but concave for species of *Euproops*. Anderson (1994) and others (e.g. Schultka 2000) have pointed out that many characters of the anterior shield are problematic for discrimination, due to their vulnerability to taphonomic deformation. Hence, size ratios, shape of shield rim, or angles of ophthalmic spines are likely to be altered under different types of preservation (see also Tashman et al. 2019). Also certain aspects of the ornament should be added here. As Haug et al. (2012) have shown, aspects of the proximal appendage parts can be preserved either ventrally or compressed through the shield and be mistaken for a kind of ornament.

It is very apparent that all specimens interpreted as representatives of *Belinurus* fall into the size groups 1–6, while all specimens interpreted as representatives of *Euproops* (or *Prestwichia* and *Prestwichianella*, which are synonyms) fall into the size groups 7–9. Together with the differences in the morphology of the structures surrounding the thoracetron—spines in specimens of *Belinurus* and a flange in specimens of *Euproops*—*Euproops* and *Belinurus* have long been accepted as two separate entities besides the phylogenetic analysis of Lamsdell (2016) which indicated that *Euproops* is in fact an ingroup of *Belinurus*.

**Fig. 12:**
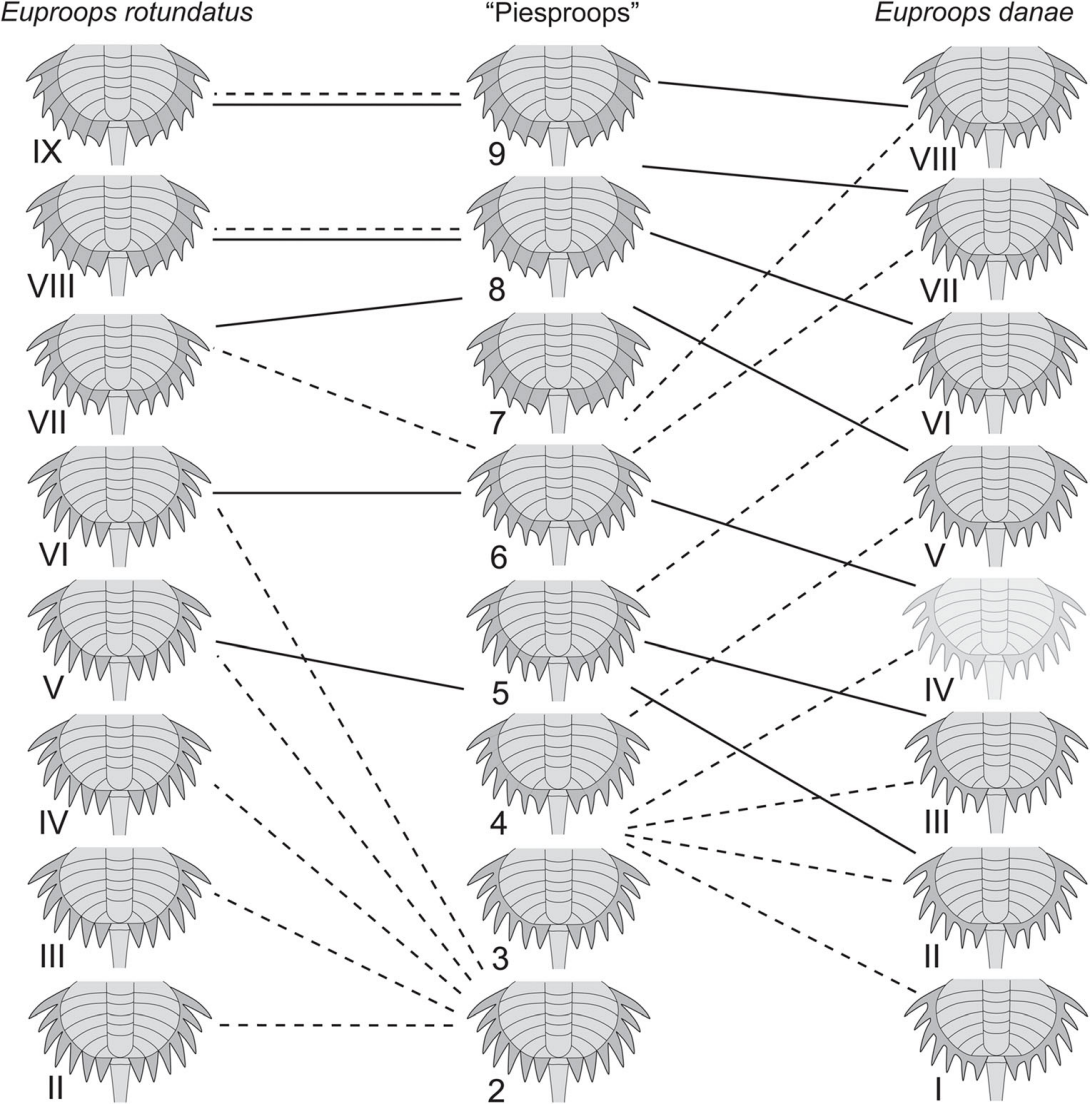
Comparison of developmental patterns. Solid lines indicate similar sizes, stippled lines similar morphologies. Note that the morphological change that occurs in the “Piesproops” from stage 2 to stage 8 happens within much fewer stages in *Euproops rotundatus*, namely from stage VI to stage VIII. Sequence of the “Piesproops” and *Euproops danae* from Haug et al. (2012) and Haug and Rötzer (2018), sequence of *Euproops rotundatus* based on the current study

Yet, due to the knowledge of the ontogeny of the “Piesproops” (representatives of *Euproops* from Germany; Haug et al. 2012), the case is in fact much less clear. The “Piesproops” has demonstrated that a flange is formed successively during post-embryonic development. It is therefore not easy to exclude the possibility that the specimens interpreted as *Belinurus* have simply not yet developed a flange. In this aspect, it is important to note that size groups 1–9 form a very gradual sequence of size gain, very comparable with the ten stages known from the “Piesproops” (Fig. 12). The nine size groups described here could therefore be well understood as representing more or less distinct stages of one ontogenetic sequence. The formation of the flange would then happen quite abruptly, with the flange missing until size group 6, being partially developed in size group 7 and fully developed from size group 8 onwards (Fig. 12).

### Consequences for xiphosurid taxonomy, part 1

The differences in size as well as the differences in thoracetron morphology cannot be used as diagnostic characters as they appear to depend on the ontogenetic stage. We therefore factually lack a clear diagnostic character for distinguishing the species of *Belinurus* from the British Coal Measures from the co-occurring species of *Euproops*. Knowing the ontogenetic sequence of the *Euproops* species from Germany as well as the one from *E. danae*, an interpretation of the morphological differences in the British species as mere ontogenetic ones seems very parsimonious.

This suggests that the specimens investigated here and originally interpreted as representatives of *Belinurus bellulus*, *B. koenigianus*, *B. baldwini*, and *B. reginae* are in fact immature representatives of *Euproops*. This would most likely make all these synonyms of species of *Euproops* (but see the next point).

### The long-spined forms

As pointed out, there are in fact two additional morphotypes: type 4, originally interpreted as representatives of *Belinurus* with longer spines, and type 5, originally interpreted as *Euproops anthrax* (Fig. 9). Type 4 could be well understood as immature form of type 5, both being representatives of *Euproops anthrax*, yet the case could be even more complicated.

The spines of specimens of type 4 appear longer than those of, for example, type 1 size class 5, which is their closest body size correspondence. Yet, this difference is largely due to the distal regions of the spines in type 4 which appears very thin and narrow. It cannot be easily excluded that this very distal part is simply not well preserved in all the other specimens of size classes 1–6. Also it cannot be simply excluded that specimens of type 5 are in fact ontogenetically further advanced forms of type 2; both possess a proximal flange. Therefore, the case may be as complicated as follows:

Types 1 and 4 could be different modes of preservation of a single morphotype of several immature stages developing into type 2. Both types 3 and 5 could be interpreted as the final morphologies of such a sequence. Finally, as the morphological differentiation of the thoracetron spines is only present in the largest stages (if the spine length in the smaller stages is only preservational), it could represent nothing else but a sexual dimorphism.

In extant xiphosurids, sexual dimorphism is well known (e.g. Loveland and Botton 1992; Lamsdell and McKenzie 2015). This sexual dimorphism is also expressed in the armature of the thoracetron, making the present case at least a plausible candidate for sexual dimorphism.

### Consequences for xiphosurid taxonomy, part 2

We can make a good argument that there are in fact fewer species of xiphosurids in the British Coal Measures than so far assumed. Specimens generally interpreted as representatives of *Belinurus* are much more likely to be immature representatives of *Euproops*. Yet, the question whether the two supposed species *E. rotundatus* and *E. anthrax* are real species or are in fact sexual dimorphs of a single species is less clear. It may therefore be premature to provide distinct synonym recognition of the two supposed species of *Euproops*. Also it may be possible that some of the distinct species of *Belinurus* are a synonym of *E. rotundatus* while others are a synonym of *E. anthrax*. We can therefore not provide a distinct synonymisation here.

It might seem unusual to interpret two species groups (“genera”) as representing ontogenetic variances of only one group. Yet this is in fact not an uncommon pattern (prominent examples from dinosaurs: Horner and Goodwin 2009; Scannella and Horner 2010). Organisms that change their morphology during ontogeny will differ from each other in different developmental stages. Without a larger sample size, such cases are difficult to identify. Only a careful comparative frame, as for example provided here, can at least provide plausible explanations (e.g. Haug and Haug, 2016, 2017).

### Consequences for xiphosurid taxonomy, part 3

With the present investigation, we have clear indications that the ontogeny of *E. rotundatus* differs from the one of the “Piesproops”. Therefore, although their adults have an identical morphology, we can see differentiations between the two. The ontogeny of the German species appears more gradual (Fig. 12). It seems therefore valid to recognise the German specimens as species separate not only from *E. danae* but also from *E. rotundatus*. A single known specimen of the little known species *E. bifidus* from a quarry close to the Piesberg, Ibbenbüren (Siegfried 1972), might be conspecific to the “Piesproops”. Yet, as apparent by the here presented case, ontogenetic data will be necessary to conclude more reliably about this issue.

### Heterochrony in evolution of the group Euproops: comparing *E. rotundatus* and the “Piesproops”

Although we face some uncertainties with the here reconstructed ontogeny of *E. rotundatus*, we have now three more or less reliable ontogenetic sequences: one for *E. rotundatus*, one for *E. danae*, and one for the “Piesproops”. The developmental pattern represented by specimens of types 4 and 5 (*E. anthrax*) is not considered here as it is too incomplete, yet the known part is partially reminiscent of the condition in *E. danae*. The relationship between the three species *E. rotundatus*, *E. danae,* and the “Piesproops” must be considered unclear.

Still, we can compare certain aspects of their developmental sequence (Fig. 12). The ontogeny of the “Piesproops” is very gradual. The developmental sequence of *E. rotundatus* includes three distinct morphologies; the transition between these is more saltatory compared with the developmental sequence of the “Piesproops”. More gradual ontogenetic sequences are usually an ancestral feature (e.g. Walossek 1993; Haug and Haug 2013; Haug et al. 2013; Haug in press). We also therefore consider the pattern as exemplified by the “Piesproops” as the more ancestral one, the one exemplified by *E. rotundatus* as derived.

By comparing details of the sequence, it becomes apparent that *E. rotundatus* retains the morphology of earlier stages (without flange) for a longer time and then “jumps” to a further derived morphology after stage VI (Fig. 12). This pattern can best be understood as cause by a combination of a developmental delay and acceleration, a pattern also seen within different crustaceans (Haug in press), including different lineages of Insecta (Haug et al. 2016). The ontogeny of *E. rotundatus* is therefore more metamorphic than that of the “Piesproops”. This kind of differentiation appears to be partly coupled to a niche differentiation between earlier and later stages (Rötzer and Haug 2015; Haug in press). We can only speculate that this might well also be the case here, but details of the life style of Carboniferous species are still very limited. Some of the suggested life styles have no modern counterparts (e.g. Fisher 1979) and remain questionable.

### Heterochrony in evolution of the group *Euproops*: comparing *E. danae* and the “Piesproops”

More complex is the case with *E. danae*. The developmental pattern of *E. danae* is “less far” than in the “Piesproops”, a specific morphology is reached later in ontogeny, the largest known stages appear more juvenile than similar-sized “Piesproops” specimens (Fig. 12). *E. danae* is therefore less metamorphic than the “Piesproops”, as the difference between early and late stages is larger in the “Piesproops”. Yet, this does not provide an evolutionary direction. The pattern of the “Piesproops” may be the more ancestral one and that of *E. danae* evolved by paedomorphosis. Paedomorphosis leads to a more juvenile appearance. There are three sub-categories of paedomorphosis (e.g. Webster and Zelditch 2005). As *E. danae* appears to reach similar adult sizes as the “Piesproops”, progenesis can be excluded, as progenesis leads to smaller final sizes. The other two sub-categories are neoteny and post-displacement. Neoteny describes a slowed down pattern, post-displacement a later started one. When comparing size and morphology of the two species (Fig. 12), there are many parallel lines connecting similar morphologies between the “Pieproops” and *E. danae*. If the pattern would be slowed down, we should see a fanning of the lines. Therefore, probably a post-displacement occurred, a later start of the development of the flange.

Yet, the case could also be the other way around. The pattern exemplified by *E. danae* could represent an ancestral one. The pattern occurring in the “Piesproops” would then have evolved by a combination of hypermorphosis and pre-displacement (Haug et al. 2010). For a reliable decision between the two possibilities, we would need a character polarisation. Yet, so far our knowledge of the ontogeny of other xiphosurids is still limited and demands for further investigations.

For the moment, we can only conclude that there are three distinct developmental patterns represented by the different species of *Euproops*. The patterns apparently evolved by heterochrony, leading to a rather metamorphic developmental pattern in *E. rotundatus*.

## References

[CR1] Anderson LI (1994). Xiphosurans from the Westphalian D of the Radstock Basin, Somerset coalfield, the South Wales Coalfield and Mazon Creek, Illinois. Proc Geol Assoc.

[CR2] Anderson LI, Selden PA (1997). Opisthosomal fusion and phylogeny of Palaeozoic Xiphosura. Lethaia.

[CR3] Battelle BA, Sombke A, Harzsch S, Schmidt-Rhaesa A, Harzsch S, Purschke G (2016). Xiphosura. Structure and evolution of invertebrate nervous systems.

[CR4] Dunlop JA, Penney D, Jekel D (2019) A summary list of fossil spiders and their relatives. In: World Spider Catalog. Natural History Museum Bern, online at http://wsc.nmbe.ch, version 19.5, accessed on 02 Sep 2019

[CR5] Fisher DC (1977). Functional significance of spines in the Pennsylvanian horseshoe crab *Euproops danae*. Paleobiol.

[CR6] Fisher DC, Nitecki MH (1979). Evidence for subaerial activity of *Euproops danae* (Merostomata, Xiphosurida). Mazon Creek fossils.

[CR7] Haug JT (in press) Chapter 9. Metamorphosis in crustaceans: towards a synthesis. In: Anger K, Harzsch S, Thiel M (eds) Developmental biology and larval ecology. The natural history of the Crustacea, vol 7. Oxford University Press, Oxford

[CR8] Haug JT, Haug C (2013). An unusual fossil larva, the ontogeny of achelatan lobsters, and the evolution of metamorphosis. Bull Geosci.

[CR9] Haug C, Haug JT, Kliman RM (2016). Developmental paleontology and Paleo-Evo-Devo. Encyclopedia of evolutionary biology.

[CR10] Haug C, Haug JT, Nuño dela Rosa L, Müller GB (2017). Methods and practices in Paleo-Evo-Devo. Evolutionary developmental biology.

[CR11] Haug C, Rötzer MA (2018). The ontogeny of the 300 million year old xiphosuran *Euproops danae* (Euchelicerata) and implications for resolving the *Euproops* species complex. Dev Genes Evol.

[CR12] Haug JT, Haug C, Ehrlich M (2008). First fossil stomatopod larva (Arthropoda: Crustacea) and a new way of documenting Solnhofen fossils (Upper Jurassic, Southern Germany). Palaeodiv.

[CR13] Haug Joachim T., Maas Andreas, Waloszek Dieter (2009). †Henningsmoenicaris scutula, †Sandtorpia vestrogothiensis gen. et sp. nov. and heterochronic events in early crustacean evolution. Earth and Environmental Science Transactions of the Royal Society of Edinburgh.

[CR14] Haug JT, Haug C, Kutschera V, Mayer G, Maas A, Liebau S, Castellani C, Wolfram U, Clarkson ENK, Waloszek D (2011). Autofluorescence imaging, an excellent tool for comparative morphology. J Microsc.

[CR15] Haug C, Van Roy P, Leipner A, Funch P, Rudkin DM, Schöllmann L, Haug JT (2012). A holomorph approach to xiphosuran evolution—a case study on the ontogeny of *Euproops*. Dev Genes Evol.

[CR16] Haug JT, Audo D, Charbonnier S, Haug C (2013). Diversity of developmental patterns in achelate lobsters—today and in the Mesozoic. Dev Genes Evol.

[CR17] Haug JT, Labandeira CC, Santiago-Blay JA, Haug C, Brown S (2015). Life habits, hox genes, and affinities of a 311 million-year-old holometabolan larva. BMC Evol Biol.

[CR18] Haug JT, Haug C, Garwood R (2016). Evolution of insect wings and development – new details from Palaeozoic nymphs. Biol Rev.

[CR19] Horner JR, Goodwin MB (2009). Extreme cranial ontogeny in the Upper Cretaceous dinosaur *Pachycephalosaurus*. PLoS One.

[CR20] Kerp H, Bomfleur B (2011). Photography of plant fossils – new techniques, old tricks. Rev Palaeobot Palynol.

[CR21] König C (1820-1825) Icones fossilium sectiles. G. B. Sowerby, London, pp 1–4 19 pls

[CR22] Lamsdell JC (2016). Horseshoe crab phylogeny and independent colonizations of fresh water: ecological invasion as a driver for morphological innovation. Palaeontology.

[CR23] Lamsdell JC, McKenzie SC (2015). *Tachypleus syriacus* (Woodward)—a sexually dimorphic Cretaceous crown limulid reveals underestimated horseshoe crab divergence times. Organisms Div Evol.

[CR24] Loveland RE, Botton ML (1992). Size dimorphism and the mating system in horseshoe crabs *Limulus polyphemus* L. Animal Behav.

[CR25] Malakhov VV (2010). A new system of Bilateria. Her Russ Acad Sci.

[CR26] Pictet FJ (1846) Traite élémentaire de paléontologie, Vol. 4. Langlois et Leclerq, Paris, 458 pp.

[CR27] Rötzer MAIN, Haug JT (2015) Larval development of the European lobster and how small heterochronic shifts lead to a more pronounced metamorphosis. Intl J Zool 2015:art 345172

[CR28] Scannella JB, Horner JR (2010). *Torosaurus* Marsh, 1891, is *Triceratops* Marsh, 1889 (Ceratopsidae: Chasmosaurinae): synonymy through ontogeny. J Vertebr Paleontol.

[CR29] Schultka S (2000). Zur Palökologie der Euproopiden im Nordwestdeutschen Oberkarbon. Mitt Mus Naturwiss Berl Geowiss R.

[CR30] Sekiguchi K (1988). Biology of horseshoe crabs.

[CR31] Selden PA, Siveter DJ (1987). The origin of the limuloids. Lethaia.

[CR32] Siegfried P (1972). Ein Schwertschwanz (Merostomata, Xiphosurida) aus dem Oberkarbon von Ibbenbüren/Westf. PalZ.

[CR33] Tashman JN, Feldmann RM, Schweitzer CE (2019). Morphological variation in the Pennsylvanian horseshoe crab *Euproops danae* (Meek and Worthen, 1865) (Xiphosurida, Euproopidae) from the lower Mercer Shale, Windber, Pennsylvania, USA. J Crust Biol.

[CR34] Walossek D (1993). The Upper Cambrian *Rehbachiella kinnekullensis* and the phylogeny of Branchiopoda and Crustacea. Foss Strata.

[CR35] Webster M, Zelditch ML (2005). Evolutionary modifications of ontogeny: heterochrony and beyond. Paleobiol.

[CR36] Williams KL, Williams KL (2019). *Limulus* as a model organism. Endotoxin detection and control in pharma, *Limulus*, and mammalian systems.

